# Lithium anode stable in air for low-cost fabrication of a dendrite-free lithium battery

**DOI:** 10.1038/s41467-019-08767-0

**Published:** 2019-02-22

**Authors:** Xiaowei Shen, Yutao Li, Tao Qian, Jie Liu, Jinqiu Zhou, Chenglin Yan, John B. Goodenough

**Affiliations:** 1 College of Energy, Key Laboratory of Advanced Carbon Materials and Wearable Energy Technologies of Jiangsu Province, Key Laboratory of Advanced Optical Manufacturing Technologies of Jiangsu Province & Key Laboratory of Modern Optical Technologies of Education Ministry of China, Soochow University, Suzhou, 215006 P. R. China; 2 Materials Science and Engineering Program and Department of Mechanical Engineering, The University of Texas at Austin, Austin, 78712 TX USA

## Abstract

Lithium metal, the ideal anode material for rechargeable batteries, suffers from the inherent limitations of sensitivity to the humid atmosphere and dendrite growth. Herein, low-cost fabrication of a metallic-lithium anode that is stable in air and plated dendrite-free from an organic-liquid electrolyte solves four key problems that have plagued the development of large-scale Li-ion batteries for storage of electric power. Replacing the low-capacity carbon anode with a safe, dendrite-free lithium anode provides a fast charge while reducing the cost of fabrication of a lithium battery, and increasing the cycle life of a rechargeable cell by eliminating the liquid-electrolyte ethylene-carbonate additive used to form a solid-electrolyte interphase passivation layer on the anode that is unstable during cycling. This solution is accomplished by formation of a hydrophobic solid-electrolyte interphase on a metallic-lithium anode that allows for handling of the treated lithium anode membrane in a standard dry room during cell fabrication.

## Abstract (web summary)

The lithium metal is a promising anode material for batteries; however, the growth of dendrite and its instability against moisture are two technical challenges. Here the authors address both issues by introducing a bifunctional layer consisting of hydrophobic graphite fluoride and lithium fluoride.

## Introduction

The organic-liquid electrolyte of the lithium-ion battery is flammable and plating of a lithium-metal anode from the liquid electrolyte is plagued by dendrite formation and growths across the electrolyte during charging to create an internal short circuit with incendiary consequences^1–5^. The carbon-host anode used to avoid metallic lithium has a low capacity and is plated by lithium (Li) metal if charged too rapidly^6^. In addition, the organic-liquid electrolyte has an electronic energy gap between the lowest- unoccupied molecular orbitals (LUMO) and the highest-occupied molecular orbitals (HOMO), *E*_*g*_ = (LUMO–HOMO) that is not matched to the Fermi level of the anode, and the LUMO is about 1.2 eV below the Fermi level of lithiated carbon, which makes mandatory a solid-electrolyte interphase (SEI) that is a Li-ion conductor between the anode and the electrolyte. The Li^+^ of the SEI is supplied by the cathode, which lowers the capacity of the cell^7–10^. Moreover, the SEI forms and dissolves during cycling, exposing fresh anode surface for further SEI formation, limiting the cycle life of the cell^11–15^. There is, therefore, a need to find a way to introduce an artificial SEI at the anode/electrolyte interface that can passivate a metallic-Li anode and also allows plating of lithium dendrite free from an organic-liquid electrolyte containing no SEI-forming additive^16–18^. The artificial SEI should be a solid electrolyte having the bottom of its conduction bond well above the Fermi level of a lithium anode during a fast charge in order to ensure the plating/stripping of a dendrite-free Li-metal anode that has a long cycle life.

Metallic Li reacts strongly with moisture and, therefore, a high-cost dry room with a relative humidity (RH) of less than 1% is used for Li-metal extrusion and the assembly of cells with a Li-metal anode^19–22^. The application of a hydrophobic SEI that is wet by metallic lithium would lower the cost of fabrication.

It has been shown that dendrite-free plating of an alkali-metal anode is possible from a solid electrolyte where the alkali metal bonds strongly to (wets) the solid electrolyte;^23^ the strong bonding between the anode and the solid electrolyte constrains the anode volume change during charge/discharge cycling to be perpendicular to the anode/electrolyte interface; this one-dimensional volume change can be accommodated by cell design.

An artificial SEI that can be applied to a Li-metal anode would form a composite membrane that protects the Li-metal anode from exposure to a humid atmosphere; the composite anode membrane can then be used in a cell assembled in ambient air, thus lowering the cost of cell assembly. Reversible stripping/plating of the metallic-Li anode would occur inside a closed cell to give an increased density of stored electric power for a given cathode^24–27^.

Herein, we use graphite fluoride (GF)^28,29^ to demonstrate this concept in full coin and round cells with a LiFePO_4_ cathode, an organic liquid-carbonate or solid polymer Li^+^ electrolyte, and a composite Li anode containing a protective layer consisting of lithium fluoride (LiF) and GF^28,29^. The obtained composite (noted as GF–LiF–Li) enables long-term stability in ambient air and prevents the fresh Li metal from contacting with organic solvents in the electrolyte during cycling owing to the hydrophobic GF–LiF layer, which can effectively stabilize the interface of the working anode. As a result, the GF–LiF–Li anode exhibits a safe and dendrite-free cycling at a current density from 1 to 10 mA cm^−2^ for a long cycle life in the Li stripping/plating experiments. Furthermore, GF–LiF–Li composite also shows excellent performance after exposure to a humid atmosphere with RH of 20–35% for over 24 h, providing a comparable specific capacity and cycling stability as fresh Li-metal anodes that have not been exposed to air.

## Results

### Synthesis and characterization

**Fig. 1:**
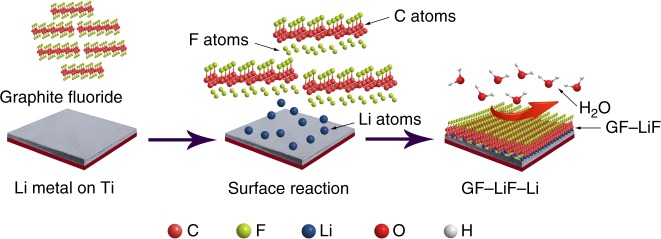
Schematic illustration of GF–LiF–Li preparation and its protective effect for Li-metal anodes. In the models, the carbon (C), fluorine (F), lithium (Li), oxygen (O), and hydrogen (H) atoms are displayed as spheres in orange, cyan, blue, red, and white, respectively

Li-metal foil was first polished to remove impurities and then heated to 250 °C on titanium foil in an argon-filled glovebox (O_2_ and H_2_O < 1 ppm) to obtain molten Li. GF powder was added slowly into the molten Li with constant stirring until the mixture was homogeneous, and then left to stand for 3 h without stirring. In a static state and at high temperature, the GF powder floats to the surface to form a uniform GF layer. The Li slowly reacted with the GF to produce LiF at the solid–liquid interface that eventually formed a GF–LiF protective layer. Once cooled, the GF–LiF–Li composite was removed from the glove box and cut into disks for assembly in coin cells. The anode membrane is flexible. Figure 1 gives a schematic illustration of the synthesis reaction.

**Fig. 2:**
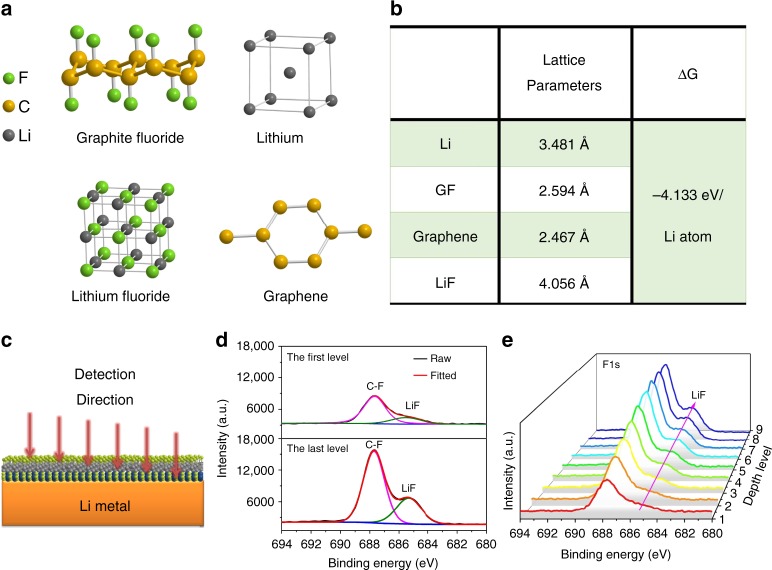
DFT calculations and XPS spectra of GF–LiF layer. **a** Optimized structures of lithium, graphite fluoride, lithium fluoride, and graphene molecular models by DFT calculations, In the models, the fluorine (F), carbon (C), and lithium (Li) atoms are displayed as spheres in green, yellow, and gray, respectively. **b** The lattice parameter of each modeling substrate. **c** The schematic diagram for the etching detection of Ar-ion sputtering. **d** High-resolution XPS F 1 s spectra of GF–LiF coating for the first and last etching level. **e** XPS depth profile of F 1 s

A detailed density functional theory (DFT) calculation using GGA-PPE computation was made to determine whether a spontaneous reaction between Li and GF is possible. For simplicity, graphene fluoride, graphene, and the unit cells of Li and LiF served as the modeling substrate to represent the reactants and products (Fig. 2a). The lattice parameters of each modeled substrate are shown in Fig. 2b. The calculations indicate that graphene fluoride is reduced to LiF and graphene spontaneously on contact with metallic Li; the Li atoms are dynamically inserted and 4.133 eV is released with every inserted Li atom. XPS analysis with Ar-ion sputtering (Fig. 2c) was conducted on the electrode to explore the surface chemistry and element spatial distribution in the GF–LiF layer. The depth profiles of the F 1 s XPS spectra are shown in Fig. 2d, e. The XPS survey spectrum of the GF–LiF–Li (Supplementary Fig. 1) confirms the chemical composition of the GF–LiF coating with the elements F, O, C, and Li. High-resolution XPS F 1 s spectra of the first and last sputtered layers of the sample are presented in Fig. 2d. In both spectra, the peaks centered at ~687.8 eV correspond to C–F bonding and the peaks at ~685.0 eV are attributed to LiF^30^, indicating that GF is the major ingredient of the GF–LiF layer. Figure 2e shows an XPS depth profile of the F 1 s spectra through the entire sputtering range. As the sputtering depth increased, the intensity of the peak corresponding to LiF increased, revealing the increasing amount of LiF in the GF–LiF coating as it approaches the Li-metal interface. In order to determine whether any fresh Li metal is exposed on the surface, an XPS measurement (area scan mode, 1.5 × 2.5 mm) was employed to investigate the GF–LiF–Li composite. The high-resolution Li 1 s spectra (Supplementary Fig. 2) did not show a peak corresponding to pristine Li metal, indicating that there is no exposure of fresh Li metal on the surface of the GF–LiF–Li composite and that the surface is well covered by the protective GF–LiF layer. To confirm the fine structure of GF–LiF–Li composite, focused-ion-beam scanning electron microscopy (FIB-SEM) tomography analysis was employed to perform a chemical characterization of the anode membrane. Supplementary Fig. 3a shows the imaged cross-section of the GF–LiF–Li composite which was trenched to a depth of 10 μm by FIB. It can be seen that GF–LiF–Li composite is composed of three layers, including the top GF–LiF layer, followed by a transitional zone consisting of GF, LiF, and Li metal and a bottom layer consisting solely of Li metal. In addition, Supplementary Fig. 3b–c shows energy-dispersive X-ray (EDX) maps of C and F elements on the FIB-ablated cross-sections that demonstrate the distribution of C and F elements in the GF–LiF–Li composite that have a downward trend from top to bottom, which is consistent with the imaged structure.

### Dendrite-free studies of the GF–LiF–Li anode

**Fig. 3:**
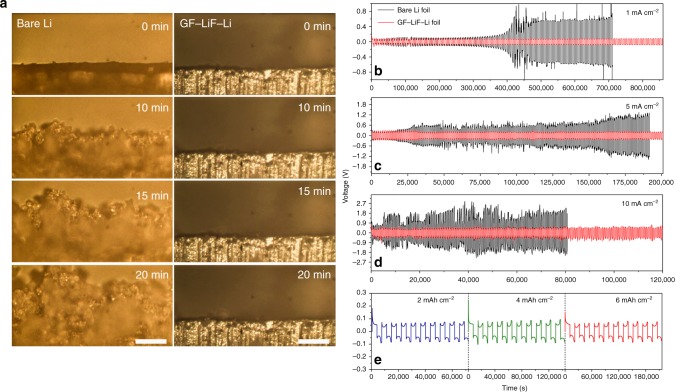
In situ optical microscopy visualization of Li electrodeposition and long-term cycling on symmetric cells. **a** The images from microscopy of the bare Li (left column) and GF–LiF–Li (right column) electrolyte interface at 0, 10, 15, and 20 min at a current rate of 3 mA cm^–2^. The scale bars are 200 μm. Corresponding movies are provided in the Supplementary information. The Li deposition of bare Li (left) and GF–LiF–Li (right) in Li plating/stripping process. Bare Li foil symmetric cells (black) and GF–LiF–Li symmetric cells (red) at various current densities of (**b**) 1 mA cm^–2^, (**c**) 5 mA cm^–2^, and (**d**) 10 mA cm^–2^. The capacity is 1 mAh cm^–2^. **e** Cycling performance of symmetric GF–LiF–Li cells cycled under 2 mA cm^−2^ with 2–6 mAh cm^−2^ conditions

Supplementary Fig. 4 shows a schematic diagram of Li deposition in the plating/stripping process on bare Li anodes where dead Li is generated and on a GF–LiF layer coated with Li anode where the formation of dendrites is suppressed. This schematic was verified with field-emission scanning electron microscopy (FESEM) characterization. The thickness change of the GF–LiF–Li anodes was measured by SEM before and after Li plating (Supplementary Fig. 5a and d). The fresh GF–LiF–Li anode showed a thickness of around 300 μm prior to the Li plating process and a thickness of about 337 μm after the Li plating process without any indication of Li dendrite formation. The corresponding EDX elemental maps (Supplementary Fig. 5b–f) indicate that F and C elements are uniformly distributed on the anode surface before and after Li plating, which reveals that the Li-metal anode remains well-protected by the GF–LiF layer as the metallic Li is plated under it. Additionally, an in situ optical microscopy visualization was conducted. A symmetric cuvette-type optical cell was fabricated to investigate the morphology of the surface of bare Li and GF–LiF–Li electrodes during the Li deposition process, which was viewed through in situ optical microscopy (see supplementary video 1 and 2). The images that are recorded at different times are displayed in Fig. 3a. The symmetric cell was subject to a high fixed current density of 3 mA cm^−2^. In the beginning, the pristine Li electrode on which Li was plated was smooth and flat, but Li dendrites appeared immediately upon imposition of the current and numerous moss-like dendrites formed on the bare Li anode as time went on. In comparison, the GF–LiF–Li electrode was seen to electrodeposit Li uniformly at high current density with a flat surface with almost no dendritic structure. This observation suggests that the GF–LiF layer can suppress the formation of Li dendrites effectively in a Li-metal rechargeable battery. Additional electrochemical performance of the GF–LiF–Li symmetric cells was studied with 1 M LiPF_6_ in 1:1 in ethylene carbonate (EC)/diethyl carbonate (DEC) (1 M LiPF_6_/EC/DEC) electrolyte at different current densities with respect to the geometric area of the working electrodes. The GF–LiF–Li cells were discharged for 1 h followed by 1 h of charge at a current density of 1 mA cm^–2^, which delivers a capacity of 1 mAh cm^–2^ with a low overpotential of around 70 mV (Fig. 3b). The voltage hysteresis (a sum of the overpotentials for Li stripping/plating^31^) shows excellent stability with a negligible voltage fluctuation during repeated cycling. In contrast, cells with bare Li foils have a much larger overpotential (~100 mV) and showed random voltage oscillations that increase during cycling. A magnified view of the voltage profiles of cells with bare Li electrodes (black) and GF–LiF–Li electrodes (red) is provided in Supplementary Fig. 6a, b, which shows that both bare Li and GF–LiF–Li anodes give a steady voltage plateau with a relatively low overpotential on the 2nd cycle. With futher cycling, the plateau of bare Li anodes becomes less smooth until a sudden voltage change occurrs at the 50th cycle, indicating that the SEI on the bare Li is continually being broken/reformed and accumulates on the surface of the Li electrodes. Cycling at elevated current densities (5and 10 mA cm^−2^), shown in Fig. 3c, d, reveals an increase in voltage hysteresis for the symmetric Li-metal cells, while the symmetric GF–LiF–Li cells exhibit a much more stable voltage profile with a smaller voltage hysteresis. The corresponding partially enlarged view of the voltage profiles (2nd and 50th cycle) at current densities of 5 mA cm^−2^ and 10 mA cm^−2^ are displayed in Supplementary Fig. 7 and Supplementary Fig. 8, respectively. Deep Li stripping/plating tests were also conducted; it was found that the symmetric GF–LiF–Li cells could function stably under a capacity as high as 6 mAh cm^−2^ with low overpotentials (Fig. 3e). These data indicate that the artificial GF–LiF coating is stable and effectively inhibits side reactions and suppresses the generation of Li dendrites under extremely fast Li plating/stripping. Subsequently, electrochemical impedance spectroscopy (EIS) analyses were performed on symmetric cells with bare Li and GF–LiF–Li electrodes with the results displayed in Supplementary Fig. 9a and b, respectively. With prolonged times of the Li plating/stripping process at a current density of 1 mA cm^−2^ and a capacity of 1 mAh cm^−2^, the impedance of the bare Li cell increased significantly, which can be ascribed to the formation of an SEI on the surface of the Li electrode. There is almost no significant change after 8 h of cycling in the batteries with GF–LiF–Li electrodes, indicating the stability of GF–LiF–Li interphase.

**Fig. 4:**
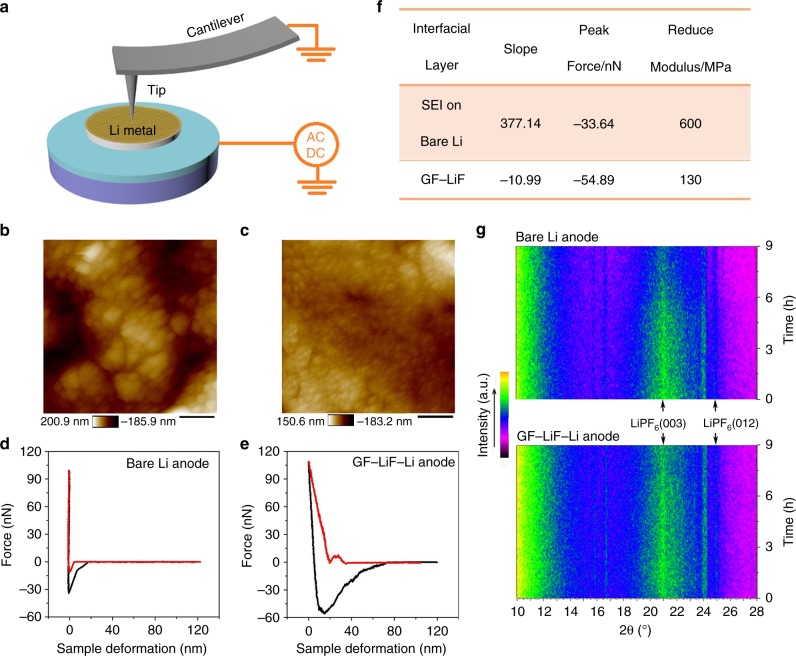
AFM and in situ XRD measurements. **a** A schematic diagram for the working principle of AFM. AFM topography images of (**b**) SEI on bare Li foil after the charging–discharging cycle and (**c**) GF–LiF–Li at room temperature. The scale bars are 400 nm. Indentation curves of (**d**) the SEI layer on bare Li and (**e**) GF–LiF layer. **f** The slope and peak force of loading curves as well as the reduced modulus. **g** The color plots represented in situ XRD patterns of bare Li anodes (top) and GF–LiF–Li anodes (bottom) upon the first charge–discharge process

Atomic force microscopy (AFM) analysis was used to show further the inhibition of Li dendrite growth by the GF–LiF–Li anodes based on a probing tip at one end of a cantilever to interact with the sample^32^. As the schematic diagram shows (Fig. 4a), both attractive and repulsive forces give rise to an interaction between the tip and the sample to give information about the topography and mechanical properties of the surface of the sample. Figure 4b shows such a topographic image of a bare Li electrode surface after cycling in 1 M LiPF_6_/EC/DEC electrolyte. The SEI layer on the Li surface is formed from reduction of the electrolyte; it gives a rough surface pitted with holes and large granular features that can provide preferential sites for the formation of dendrites and dead Li. In contrast, the surface of GF–LiF–Li is relatively smooth with the uniform coating depicted in Fig. 4c. Further studies of the morphological evolution of bare Li and GF–LiF–Li were conducted by observing the surface of the two different anodes before and after 30 charge/discharge cycles. Supplementary Fig. 10a shows the surface topography of pristine Li metal before cycling, revealing a smooth and flat surface. After 30 cycles (Supplementary Fig. 10b), Li dendrites develop on the surface of the pure Li metal, which give it a rough texture with mossy and rugged hilly sites. Supplementary Fig. 11a is the surface of a GF–LiF–Li anode with uniformly distributed nanoparticles. The smooth surface does not show any evidence of Li dendrites after cycling and the morphology of the GF–LiF–Li is maintained (Supplementary Fig. 11b), indicating that the GF–LiF coating is an ideal protective layer to suppress Li dendrites. Figure 4d, e gives the force curves as a function of tip–surface distance during the indentation loading and unloading cycle; the slopes of the load and unload curves are attributed to the stiffness of the material being probed with the AFM tip. The measured largest negative force is the adhesion force between the probed surface and tip^33^. In Fig. 4d, the loading and unloading curves of the SEI on bare Li almost overlap. The high slope of the curve and negligible viscoelasticity suggest that the SEI layers are stiff and brittle. This observation is in stark contrast to the curves of the GF–LiF–Li layer (Fig. 4e), which show a large hysteresis between the load and unload with a long pull-off or meniscus before the AFM tip completely separates from the GF–LiF–Li surface and returns to its normal position. The peak force and slope obtained from the loading curves are displayed in Fig. 4f for convenience. These data indicate that the GF–LiF layer is more elastic than its bare Li-metal counterpart. The reduced modulus of the SEI and GF–LiF surface layers was estimated with indentation testing. The modulus of the SEI layers on bare Li is tested as 600 MPa, but the GF–LiF layer delivers a low modulus of approximately 130 MPa. The decreased modulus of the GF–LiF layer suggest that it is much more flexible and not as easily broken as the SEI layers that develop on bare Li metal during the plating/stripping process.

### In situ cycling XRD study of the avoidance of electrolyte decomposition

In situ galvanostatic cycling XRD was employed to monitor the GF–LiF–Li electrodes during the first charge–discharge process in real time to determine the phase composition based on X-ray scattering^34^. Figure 4g shows that the diffraction pattern of the GF–LiF–Li electrode is stable without significant change to any diffraction peaks upon charge/discharge. However, the diffraction peaks ascribed to the LiPF_6_ (003) and (012) planes (PDF no. 52–0488) gradually weaken in the color plots of the XRD patterns of the bare Li anode used for comparison under the same testing conditions as with the GF–LiF–Li anode. The corresponding detailed XRD patterns are provided in Supplementary Fig. 12, which displays a consistent phenomenon with the color plots of the XRD patterns (Fig. 4g). The single first-scan XRD patterns of bare Li and GF–LiF–Li anodes are presented in the inset of Supplementary Fig. 12a and b, respectively. This result demonstrates the decomposition of the electrolyte during the first charge/discharge cycle of bare Li due to the formation of an SEI layer and Li dendrites. The findings of the in situ XRD study reinforce our previous assertions of the avoidance of electrolyte decomposition at the anode, as well as the lack of side reactions between Li metal and an organic-liquid electrolyte when the GF–LiF layer is present.

### Electrochemical properties

**Fig. 5:**
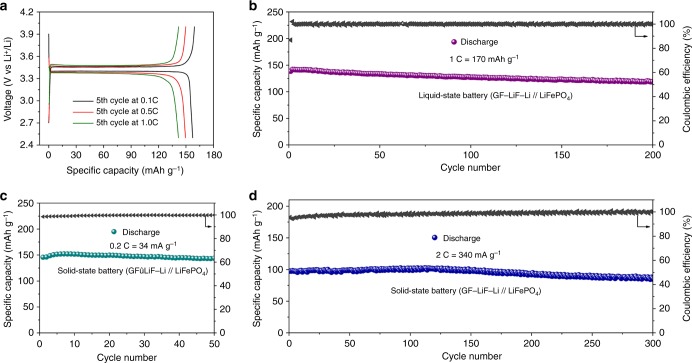
Galvanostatic cycling performance of GF–LiF–Li anodes. Characteristic charge–discharge voltage profiles of (**a**) liquid-state GF–LiF–Li/LiFePO_4_ cells at different current densities. The cycling performance of (**b**) liquid-state GF–LiF–Li/LiFePO_4_ cells. Cycling stability of all-solid-state GF–LiF–Li/LiFePO_4_ cells at a current rate of (**c**) 0.2 C and (**d**) 2 C

A high reversible capacity and good cycling stability are necessary for a battery. The electrochemical performance of GF–LiF–Li electrodes was evaluated with several different cathode and electrolyte combinations through galvanostatic cycling. Full cells with a GF–LiF–Li anode were tested at room temperature with LiFePO_4_ as the cathode and 1 M LiPF_6_/EC/DEC as an electrolyte. Figure 5a presents the voltage profiles of the fifth cycle in the voltage window of 2.5–4.0 V at various current densities. Typical discharge plateaus of LiFePO_4_ at around 3.4 V versus Li^+^/Li have a capacity of 160 mAh g^−1^, 150 mAh g^−1^, and 140 mAh g^−1^ at current rates of 0.1 C, 0.5 C, and 1 C (1 C = 170 mA g^−1^), respectively. Figure 5b shows the electrochemical performance of GF–LiF–Li/LiFePO_4_ cells with a liquid electrolyte at a current rate of 1 C; a specific capacity of 140 mAh g^−1^ with ~100% coulombic efficiency was stable for 200 cycles. Figure 5c displays the cycling performance of a GF–LiF–Li/LiFePO_4_ cell with a solid-state polymer electrolyte. The all-solid-state cell demonstrated a reversible capacity of 150 mAh g^−1^ and a high coulombic efficiency of 99.8% at 0.2 C. At 2 C (Fig. 5d), the cells with GF–LiF–Li electrodes exhibited a capacity of 102 mAh g^−1^ and outstanding cyclability, remaining stable for 300 cycles. Further charge–discharge measurements of GF–LiF–Li electrodes were taken with LiNiCoMnO_2_ as a cathode (GF–LiF–Li/LiNiCoMnO_2_). The characteristic fifth charge–discharge voltage profiles at different current densities in the voltage window of 3.0–4.3 V are shown in Supplementary Fig. 13. Where discharge plateaus around ~3.6–3.7 V are observed while the cells deliver a capacity of approximately 160 mAh g^−1^, 150 mAh g^−1^, and 140 mAh g^−1^ corresponding to current densities of 27.8 mA g^−1^, 139 mA g^−1^, and 278 mA g^−1^, respectively. The long cycle life performance of GF–LiF–Li/LiNiCoMnO_2_ cells at a high current rate of 278 mA g^−1^ is shown in Supplementary Fig. 14. The cell showed a discharge capacity of 140 mAh g^−1^ with a coulombic efficiency of around 100% corresponding to a capacity fade rate of only 0.06% per cycle for 300 cycles. The full cells with stable electrochemical performance confirm the practicability of GF–LiF–Li electrodes in a Li-metal battery system with a liquid or solid-state electrolyte.

### Air stability of GF–LiF–Li anodes

**Fig. 6:**
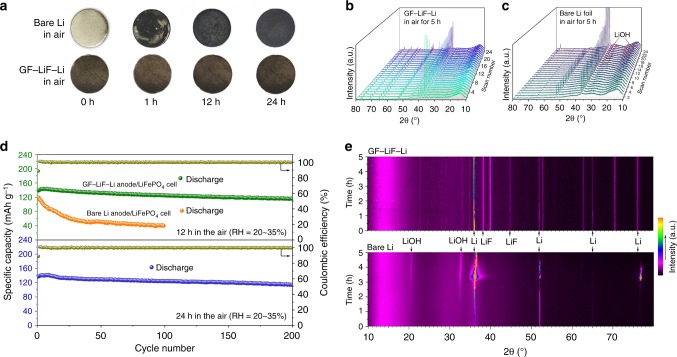
Characterization of GF–LiF–Li air stability. **a** Photographs of pristine Li and GF–LiF–Li exposed to air with relative humidity of 20–35% for various times. All in situ XRD patterns of (**b**) pristine Li metal and (**c**) GF–LiF–Li at every scan. **d** The cycling performance of anodes after an exposure in air for 12 h (top) and 24 h (bottom). **e** The color plots of in situ XRD patterns of GF–LiF–Li (top) and bare Li (bottom) in air for 5 h

We studied the stability of the anodes in ambient air; Fig. 6a shows optical photographs of the as-obtained GF–LiF–Li and bare Li foil exposed to air with RH of 20–35% for varying amounts of time. In an inert atmosphere, the pristine Li initially exhibits a silver-like color with a flat surface, but color change occurs immediately as soon as the Li foil is exposed to ambient conditions. After exposure to ambient air for 1 h, the color of Li metal turns completely ash black with a rough texture. GF–LiF–Li anodes show no significant change in color, shape, or texture for the duration of its exposure to humid air, revealing that the GF–LiF coating can serve as an excellent hydrophobic protection layer to stabilize Li metal in ambient air. The hydrophobicity of the GF–LiF coating was further investigated with in situ XRD measurements in order to analyze the entire oxidation process. The surface of the Li foil was not polished, and the RH of the test chamber was around 10%, which slowed down the rate of corrosion. The first XRD scan patterns between the bare Li and GF–LiF–Li are given in Supplementary Fig. 15 after they were just exposed to air. Bare Li and GF–LiF–Li both exhibit sharp diffraction peaks corresponding to metallic Li (PDF no. 15–0401), but the GF–LiF–Li pattern shows additional diffraction peaks of LiF (PDF no. 89–3610) that derive from the GF–LiF coating and other diffraction peaks attributed to LiC (PDF no. 14–0649), LiC_24_(PDF no. 35–1047), and Li_2_O_2_ (PDF no. 09–0355). Supplementary Fig. 16 shows the last XRD patterns conducted after 5 h of exposure. New diffraction peaks indexed to LiOH (PDF no. 32–0564) can be found in the bare Li pattern, whereas the XRD patterns of GF–LiF–Li show no obvious change. In situ XRD measurements reveal real-time observations of a continuous variation of LiOH generation as time goes on, which can be ascribed to the reaction between ambient H_2_O and Li metal (Fig. 6c for bare Li). All peaks of bare Li shifted different degrees and increase in intensity abruptly due to a shape change of Li foil that was caused by reaction with O_2_ and H_2_O in air. In contrast, there is no obvious change in the GF–LiF–Li XRD pattern over the course of its exposure to ambient conditions (Fig. 6b). The corresponding contour plots of in situ XRD patterns are displayed in Fig. 6e. The top contour represents GF–LiF–Li, and the bottom contour is for bare Li. XRD peaks associated with a LiOH phase appear in the bare Li plot and gradually increase in intensity. These findings indicate that the GF–LiF layer effectively inhibits the reaction between H_2_O and Li metal and can serve as a robust protective coating on the surface of metallic Li. Moreover, the surface chemical composition of GF–LiF–Li composites after exposure to air for 1 and 5 h was investigated to compare with fresh composites by XPS measurements. From Supplementary Fig. 17a–d, no significant changes are observed in a survey of Li 1 s, F 1 s, and C 1 s spectra, revealing the stability of GF–LiF–Li composite in air.

The feasibility of the air-stable GF–LiF–Li anodes for use in practical applications was demonstrated by galvanostatic charge/discharge cycling. Evaluation of the charge-storage capabilities of GF–LiF–Li anodes that were exposed to ambient air for 12 h, and 24 h, respectively, was conducted with cells having LiFePO_4_ as the cathode (Fig. 6d). Both of the cells with GF–LiF–Li electrodes after 12 and 24 h of air exposure presented almost the same electrochemical performance at 1 C (i.e., ~140 mAh g^−1^) as the fresh GF–LiF–Li anode (Fig. 5b). Cells with bare Li metal placed in air for 12 h were also tested in the same conditions as the GF–LiF–Li anodes for comparison. The bare Li-metal cells exhibit poor cycling stability that falls rapidly to a specific capacity of only 40 mAh g^−1^ after 70 cycles. After 24 h of exposure to ambient air, the Li metal is completely corroded and does not deliver any capacity after cell assembly in the charge/discharge process. Symmetric cells were assembled with GF–LiF–Li anodes after exposure to air to further test their electrochemical performance. Supplementary Fig. 18 presents typical electrochemical stripping curves for bare Li and GF–LiF–Li anodes; the cells were all measured at a current density of 1 mA cm^−2^. The bare Li anode delivers a specific capacity of 3616 mAh g^−2^, and GF–LiF–Li anodes before and after exposure to air for 12 h and 24 h are able to show specific capacities of 2924, 2903, and 2889 mAh g^–1^, respectively. This result indicates that ∼81 wt% of Li in the fresh GF–LiF–Li composite is active and that very little Li metal is sacrificed in the humid air. Similarly, from the voltage profiles and EIS measurements at 1 mA cm^−2^, the cycling performance and interphase stability of GF–LiF–Li anodes exposed to air for 12 and 24 h perform as well as unexposed composites (Supplementary Fig. 19 and Supplementary Fig. 20). These observations indicate that the GF–LiF–Li anodes offer an anode for Li-metal liquid-electrolyte batteries that does not require an extremely inert atmosphere for cell assembly; the practicability of this anode can effectively reduce the cost of cell fabrication.

## Discussion

A metallic-lithium anode on which dendrite-free lithium can be plated/stripped rapidly at a relatively low impedance from either a liquid or a solid electrolyte has been demonstrated in coin cells. The anode is fabricated in a glove box by incorporating GF in molten lithium at 250 °C. The Li bonds strongly with the GF to form a LiF layer, and the hydrophobic GF–LiF SEI protects the Li metal from reaction with moist air. The GF–LiF–Li composite can be removed from the glove box and assembled in a cell in ambient air. The GF–LiF composite is stable on contact with an organic liquid-carbonate electrolyte and bonds with metallic- lithium plating of a dendrite-free Li-metal anode on the GF–LiF–Li composite which grows the Li-metal anode perpendicular to the anode/electrolyte interface within a closed cell. The ethylene–carbonate additive to the liquid electrolyte conventionally used to form an SEI on a Li or lithiated-carbon electrolyte can be removed. The GF–LiF–Li composite anode can reduce fabrication costs, enable a fast charge, increase cycle life, and increase the density of stored electric power in a safe rechargeable lithium battery.

## Methods

### Synthesis of GF–LiF–Li electrodes

Lithium- (Li) metal foil was first polished to remove any impurities on the surface of the foil. The Li metal then was heated over 250 °C on titanium foil in an Ar-filled glovebox (O_2_ < 0.1 ppm and H_2_O < 0.1 ppm) to obtain a molten Li liquid. Subsequently, the graphite fluoride (GF) powder was slowly added into the molten Li (1:8 mass ratio) with constant stirring until the mixture was homogeneous, and then left to stand for 3 h to form an even GF–LiF layer on the surface of the Li metal. The final product was cooled to room temperature and was cut into disks in ambient air to obtain GF–LiF–Li anodes.

### General characterization

SEM observations were obtained using a field-emission scanning electron microscopy (FESEM, SU8010, Japan). The fine structure of the GF–LiF–Li composites was obtained by tomography analyses of focused-ion-beam scanning electron microscopy (FIB-SEM, Scios, FEI). Atomic force microscopy (AFM, Dimension Icon, BRUKER) was used to investigate the surface morphology and analyze the mechanical properties of the SEI layer on bare Li and on the GF–LiF layer. The reduced modulus of surface layers was obtained in peak force QNM mode with sharp AFM tips (BRUKER RTESPA-150). The topographic images of the bare Li SEI layer and the GF–LiF layer were recorded using tapping-mode imaging with sharp AFM tips (BRUKER RTESPA-150). The scan area size was for AFM 2 × 2 μm. The in situ surface viewed on bare Li and GF–LiF–Li electrodes was conducted in a metallurgical microscope (Caikon Optical Instrument DMM-330C) with 8.9-mm extra-long working distance 10× objectives. Surface elemental analysis was performed with X-ray photoelectron spectroscopy (XPS). Measurements were conducted in an ultra-high-vacuum ESCALAB 250 setup equipped with a monochromatic Al Kα X-ray source (1486.6 eV; anode operating at 15 kV and 20 mA). In situ X-ray diffraction investigations were recorded with an X-ray powder diffractometer (D8 ADVANCE, Bruker AXS GmbH Co., Ltd).

### Electrochemical characterization

In total, 2025-type coin cells were assembled in an Ar-filled glovebox with bare Li and GF–LiF–Li anodes, respectively. Celgard 2400 was used as the separator in all cells with 1 M LiPF_6_ in 1:1 in ethylene carbonate (EC)/diethyl carbonate (DEC) (1 M LiPF_6_/EC/DEC) as the electrolyte. Cathodes were composed of either LiFePO_4_ or LiNiCoMnO_2_ as the active material and were loaded with 80% active material, 10% acetylene, black, and 10% polyvinyldene fluoride (PVDF). The mass loading of the active materials in the cathodes was 10–14 mg cm^–2^. Galvanostatic charge–discharge tests were measured in a voltage window of 2.5–4.3 V (LiNiCoMnO_2_) and 2.5–4 V (LiFePO_4_) vs. Li^+^/Li using a battery-testing system (LAND CT 2001A, Wuhan, China). The cyclic voltammetry (CV) and electrochemical impedance spectroscopy (EIS) measurements were performed on a CHI660C electrochemical workstation in a potential window of 2.5–4.0 V (vs. Li^+^/Li) at 0.1–0.5 mV s^–1^ and in a frequency range of 0.001 Hz–100 kHz, respectively. The solid-state polymer electrolyte was prepared through a modified route following the work of Cui^35^. The solid electrolyte films were synthesized on a polypropylene membrane at 80 °C for 4 h by in situ thermal polymerization of a precursor, which was composed of 1.25 wt% lithium difluoro (oxalato) borate (LiDFOB) and 20 wt% lithium bis (trifluoromethane sulfonimide) (LiTFSI) in 20 μL of poly (ethylene glycol) diglycidyl ether (PEGDE). Symmetric cells with bare Li metal or GF–LiF–Li anodes were fabricated with 40 μL of 1 M LiPF_6_/EC/DEC without any additives.

### Computational methods

All density functional theory (DFT) calculations were performed with PWSCF code of the Quantum Espresso suite^36^. We employed ultrasoft pseudopotentials generated with the Vanderbilt recipe4 and the Perdew–Burke–Ernzerhof (PBE) approximation^37^ to the exchange-correlation function. The electronic wave functions were expanded as plane waves with an energy cutoff of 40 Ry, while with the charge density, the energy cutoff is taken to 200 Ry. For pristine graphene and graphite fluoride (GF) monolayers, hexagonal primitive unit cells were employed in the DFT calculations and a 20.0 Å of vacuum in the normal direction of the atomic plane was included to decouple periodic images. The most stable structures of bulk lithium and LiF were calculated to be body-centered cubic (BCC) and face-centered cubic (FCC), respectively, and these were used for further calculations. Brillouin-zone integrations were approximated using the special k-point sampling of Monkhorst–Pack scheme6 with a centered grid. The k-grid size was 24 × 24 × 1, 24 × 24 × 1, 20 × 20 × 20, and 20 × 20 × 20 for PG, FG, Li, and LiF, respectively. The atomic positions and lattice parameters of PG, FG, Li, and LiF were optimized and the residual force and stress in the optimized geometry are less than 0.01 eV/Å and 10^−3^ GPa, respectively. The obtained lattice parameters of PG, FG, Li, and LiF are 2.467 Å, 2.594 Å, 3.481 Å, and 4.056 Å, respectively.

## Supplementary information

Supplementary Information

Description of Additional Supplementary Files

Supplementary Movie 1

Supplementary Movie 2

## Data Availability

The data that support the findings of this study are available from the corresponding author upon reasonable request.
