# Supplementary material for: Lithium anode stable in air for low-cost fabrication of a dendrite-free lithium battery
Source: Nat Commun. 2019 Feb 22;10:900. doi: 10.1038/s41467-019-08767-0 (PMC6385276; doi:10.1038/s41467-019-08767-0)
Supplement: Supplementary file 1 — Supplementary Information [file 41467_2019_8767_MOESM1_ESM.pdf]

Supplementary Information

Lithium Anode Stable in Air for Low-Cost Fabrication of a Dendrite-Free Lithium Battery

Shen et al.

Supplementary Figures

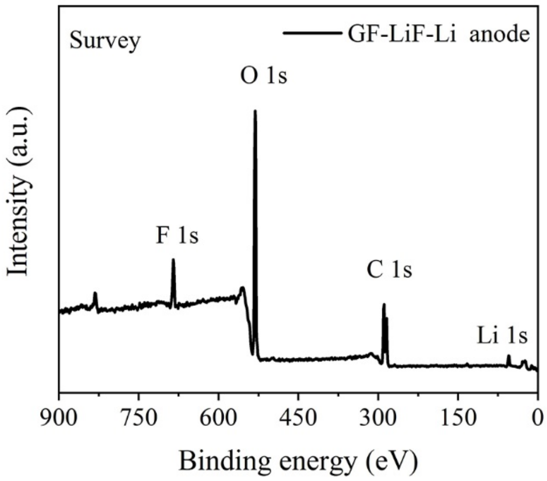

Supplementary Figure 1. The surface survey XPS spectrum of GF-LiF-Li.

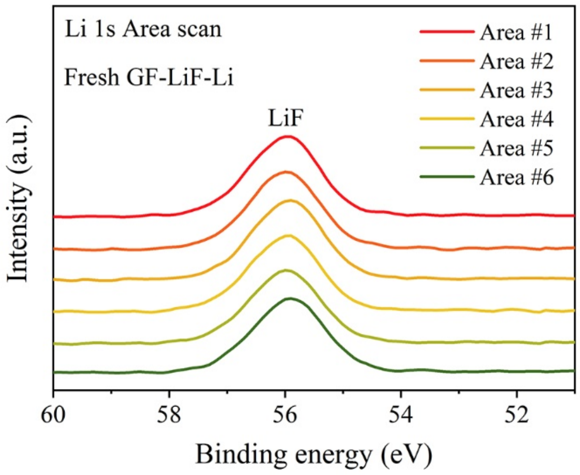

Supplementary Figure 2. High-resolution XPS Li 1s spectrum of GF-LiF-Li composite under area scan mode. The selected area is 1.5 × 2.5 mm.

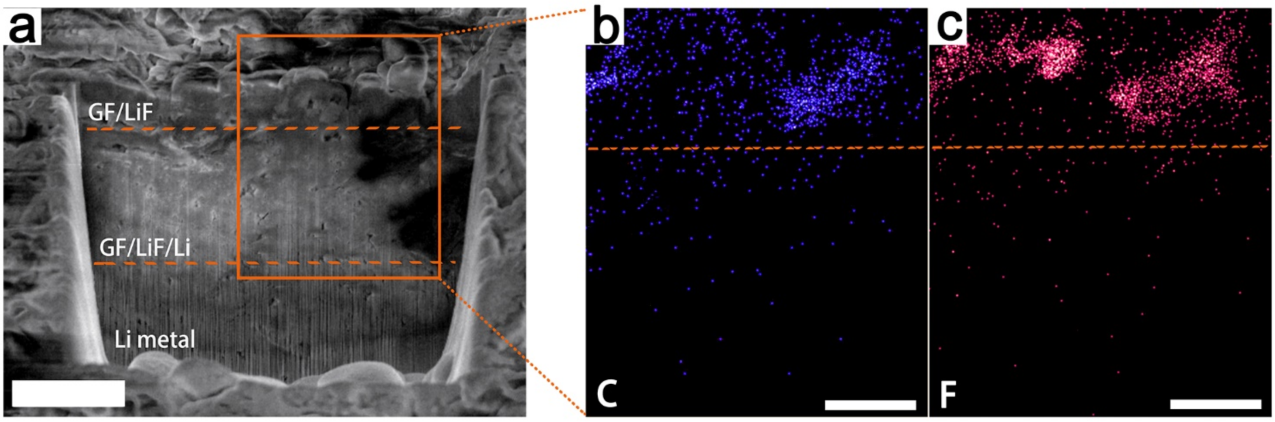

Supplementary Figure 3. FIB-SEM analysis of GF-LiF-Li composite. **a** SEM image (scale bar, 5 μm) of GF-LiF-Li cross-section carved with a FIB. The yellow frame suggests a central region within the cross-section that was selected for further analysis. **b, c** EDX maps of C and F elements. Scale bars, 2 μm.

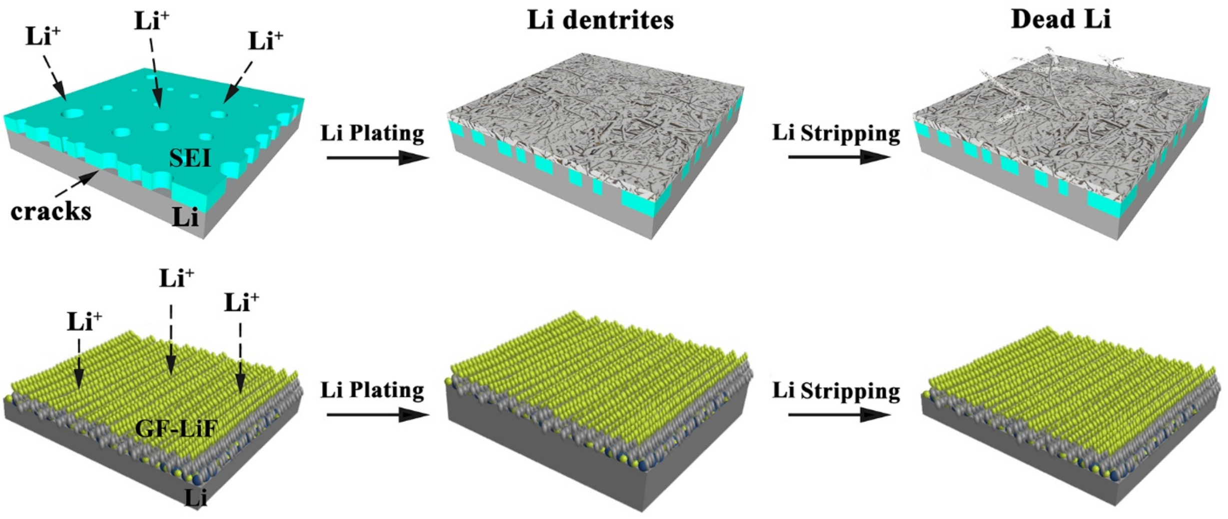

Supplementary Figure 4. The schematic diagram of Li plating/stripping during cycling.

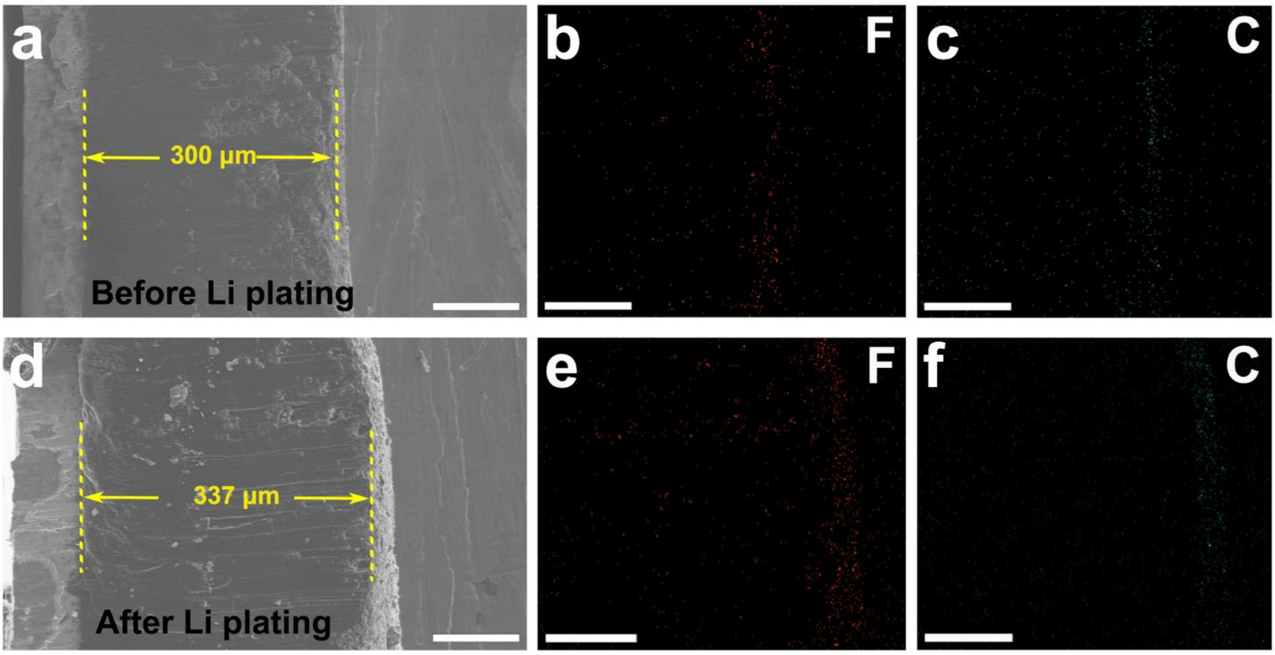

Supplementary Figure 5. Characterizations of GF-LiF-Li anode. Cross-section SEM images of GF-LiF-Li anodes (**a**) before and (**d**) after Li plating. **b–c**, **e–f** Corresponding EDS mapping of F, and C. Scale bars, 100 μm.

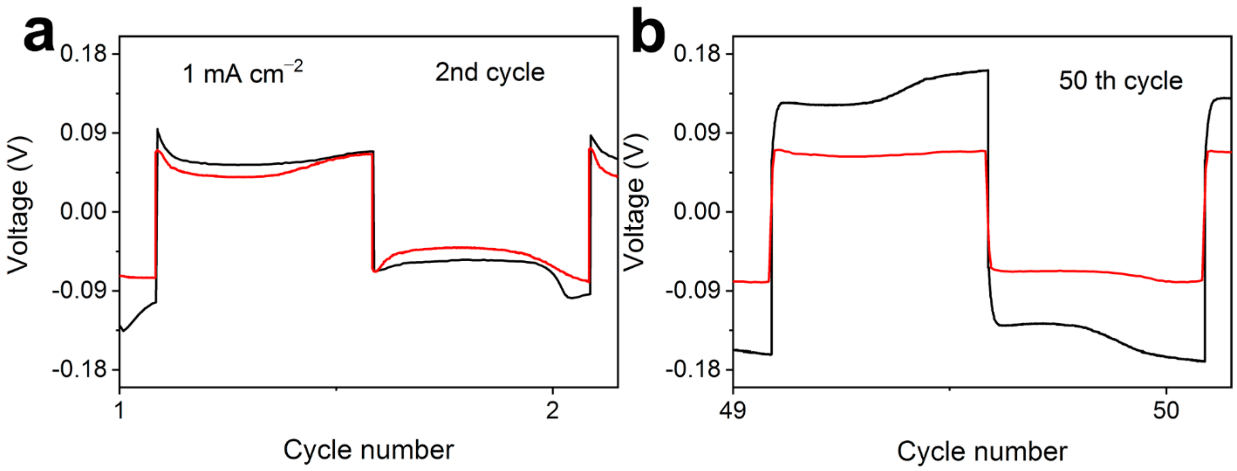

Supplementary Figure 6. Electrochemical characterizations of GF-LiF-Li electrode. Zoomed-in view of the voltage profiles of cells with bare Li electrodes (black) and GF-LiF-Li electrodes (red) (**a**) at the 2nd cycle and (**b**) the 50th cycle at the current rate of 1 mA cm^{-2}.

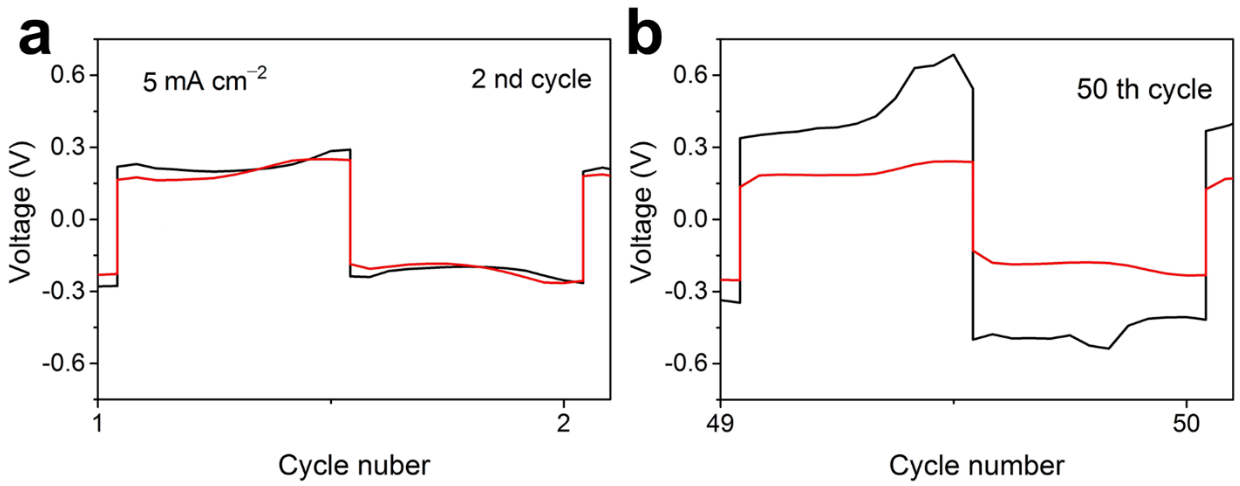

Supplementary Figure 7. Electrochemical characterizations of GF-LiF-Li electrode. Zoomed-in view of the voltage profiles of cells with bare Li electrodes (black) and GF-LiF-Li electrodes (red) (**a**) at the 2nd cycle and (**b**) the 50th cycle at the current rate of 5 mA cm^{-2}.

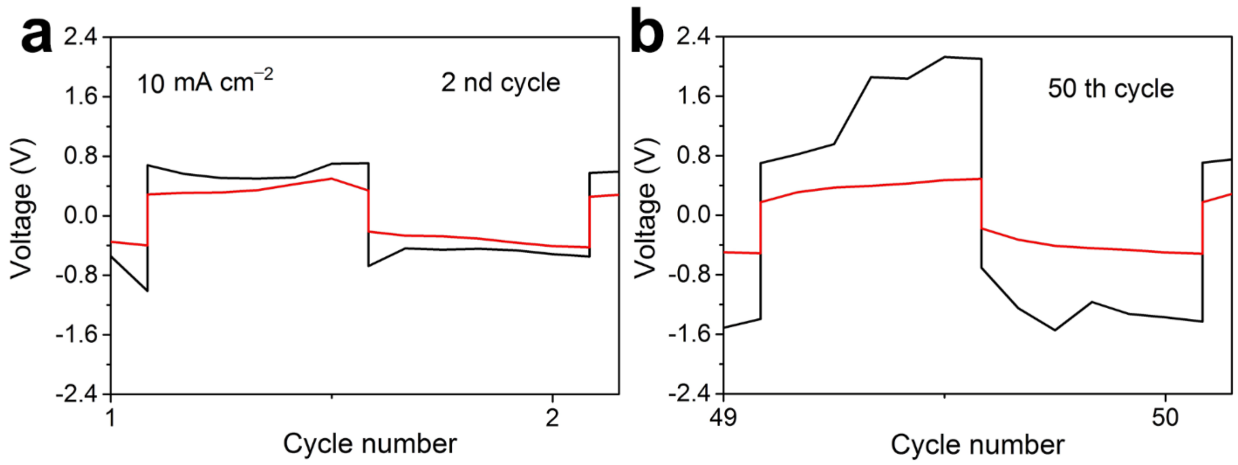

Supplementary Figure 8. Electrochemical characterizations of GF-LiF-Li electrode. Zoomed-in view of the voltage profiles of cells with bare Li electrodes (black) and GF-LiF-Li electrodes (red) (**a**) at the 2nd cycle and (**b**) the 50th cycle at the current rate of 10 mA cm^{-2}.

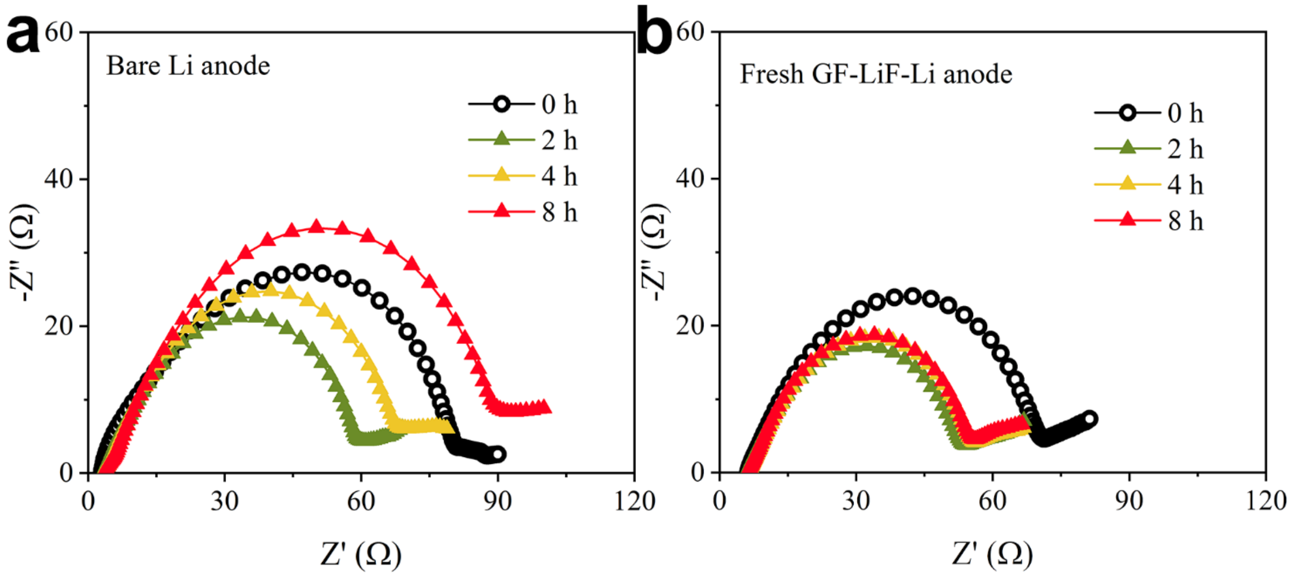

Supplementary Figure 9. EIS analysis. Time-dependent impedance spectra of symmetric batteries with (**a**) bare Li and (**b**) GF-LiF-Li electrodes before and after Li plating/string process for different durations.

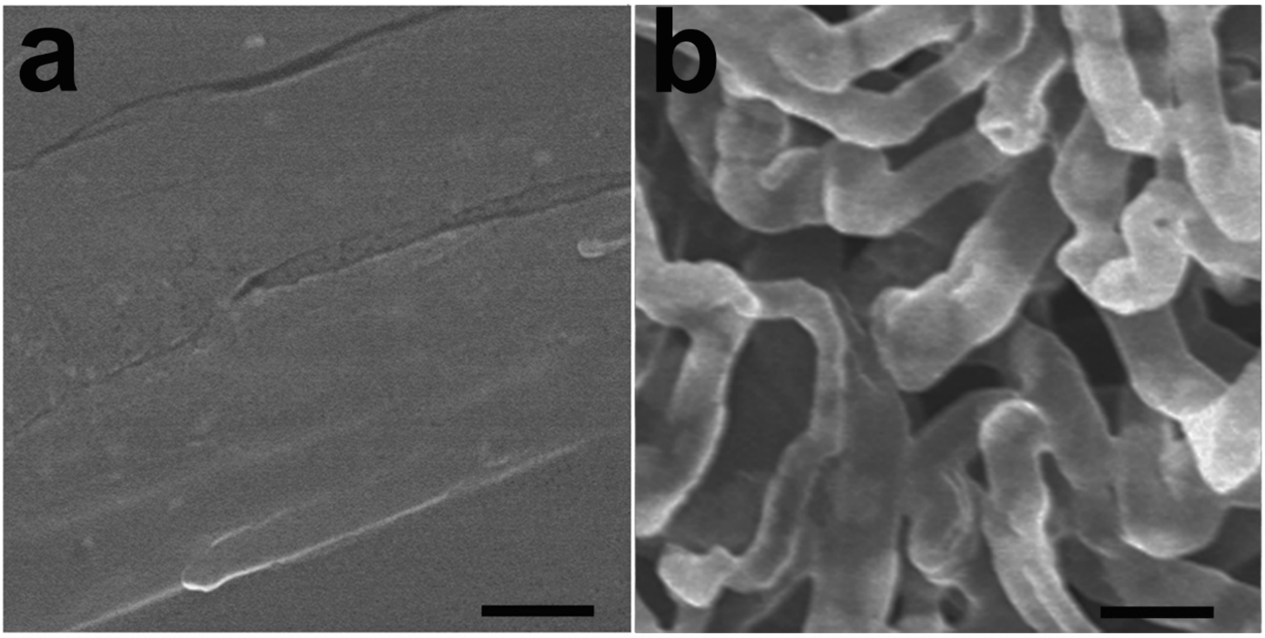

Supplementary Figure 10. SEM images of bare Li electrode. The morphology of bare Li anode (**a**) before and (**b**) after cycling. Scale bars, 500 nm.

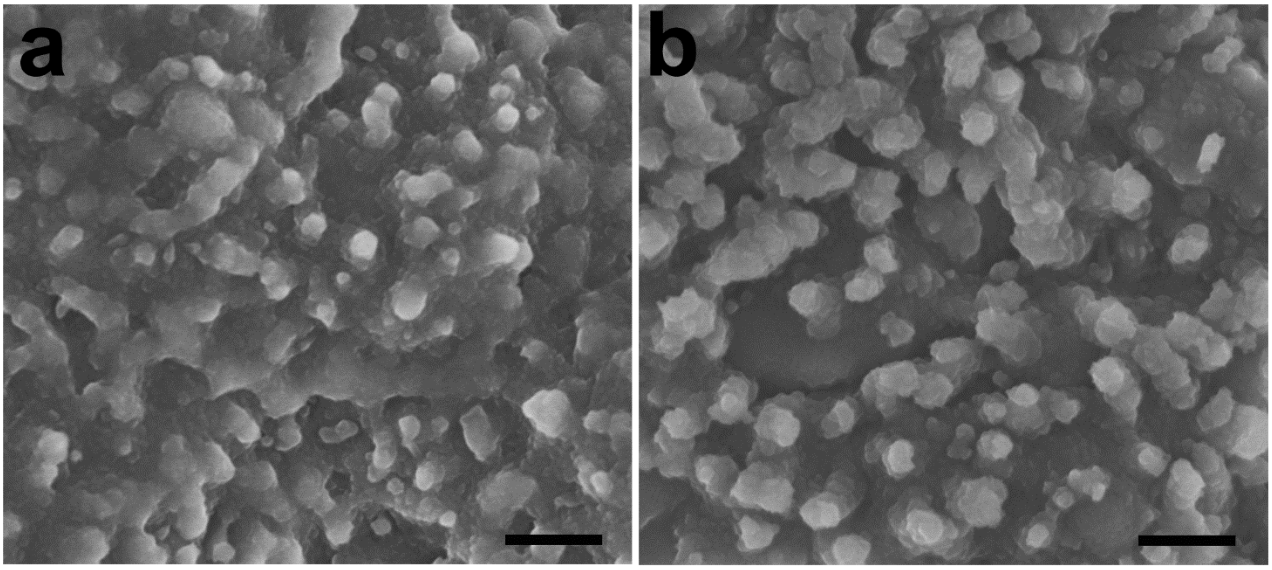

Supplementary Figure 11. SEM images of GF-LiF-Li electrode. Surface views of GF-LiF-Li anode (**a**) before and (**b**) after cycling. Scale bars, 500 nm.

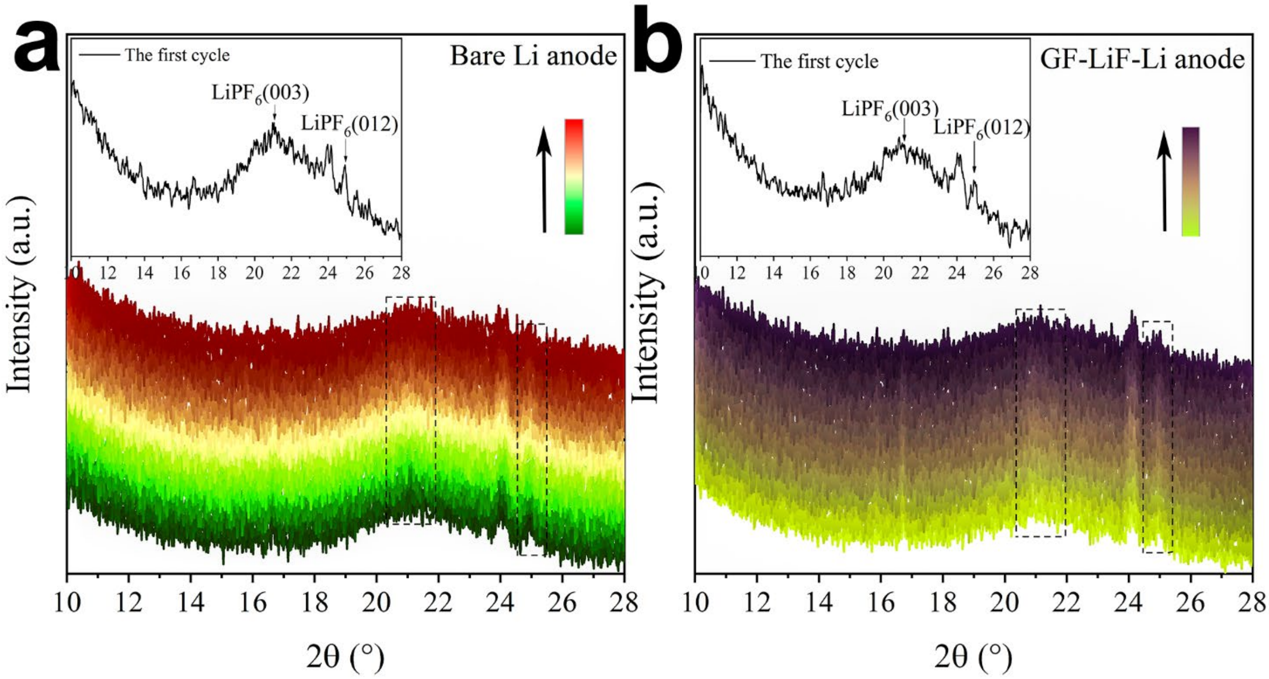

Supplementary Figure 12. *In-situ* XRD measurements. Representative *in-situ* XRD patterns of (**a**) bare Li metal and (**b**) GF-LiF-Li anode at every scan.

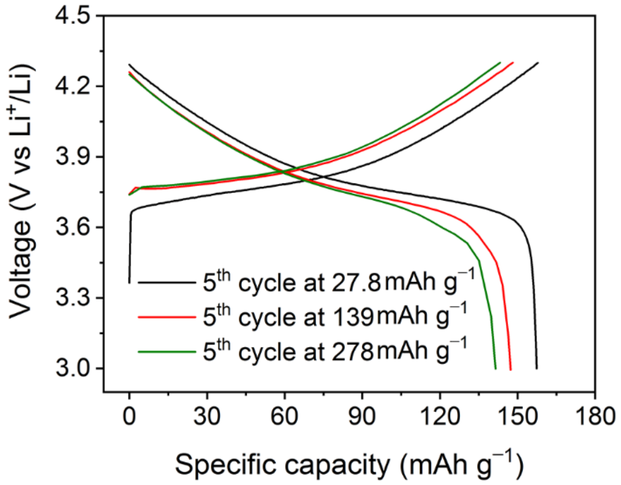

Supplementary Figure 13. The characteristic charge-discharge voltage profiles of liquid-state GF-LiF-Li//$LiNiCoMnO_2$ cells at diffierent current densities.

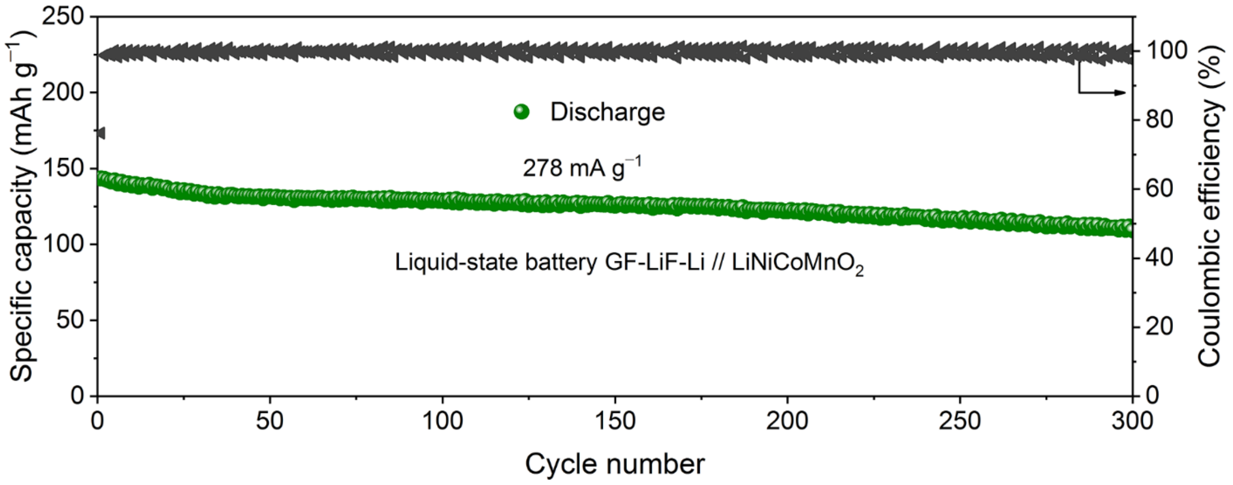

Supplementary Figure 14. The long-term cycling test of GF-LiF-Li//$LiNiCoMnO_2$ cells at a current density of 278 mA g^{-1}.

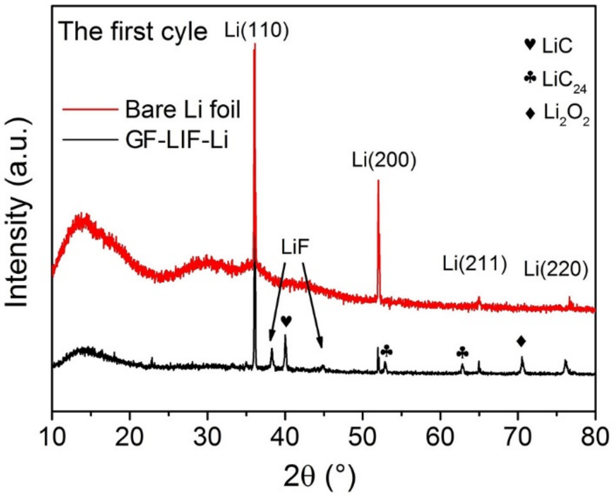

Supplementary Figure 15. Material characterizations of GF-LiF-Li. The first scan of *in-situ* XRD patterns between the pristine Li and GF-LiF-Li in air.

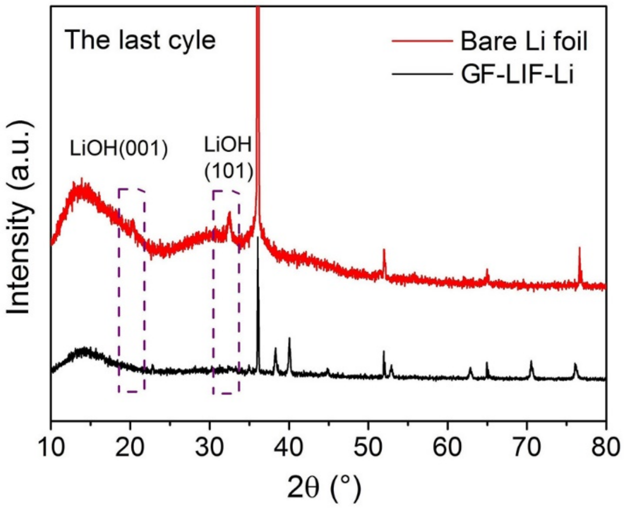

Supplementary Figure 16. Material characterizations of GF-LiF-Li. The last scan of XRD patterns between the pristine Li and GF-LiF-Li in air.

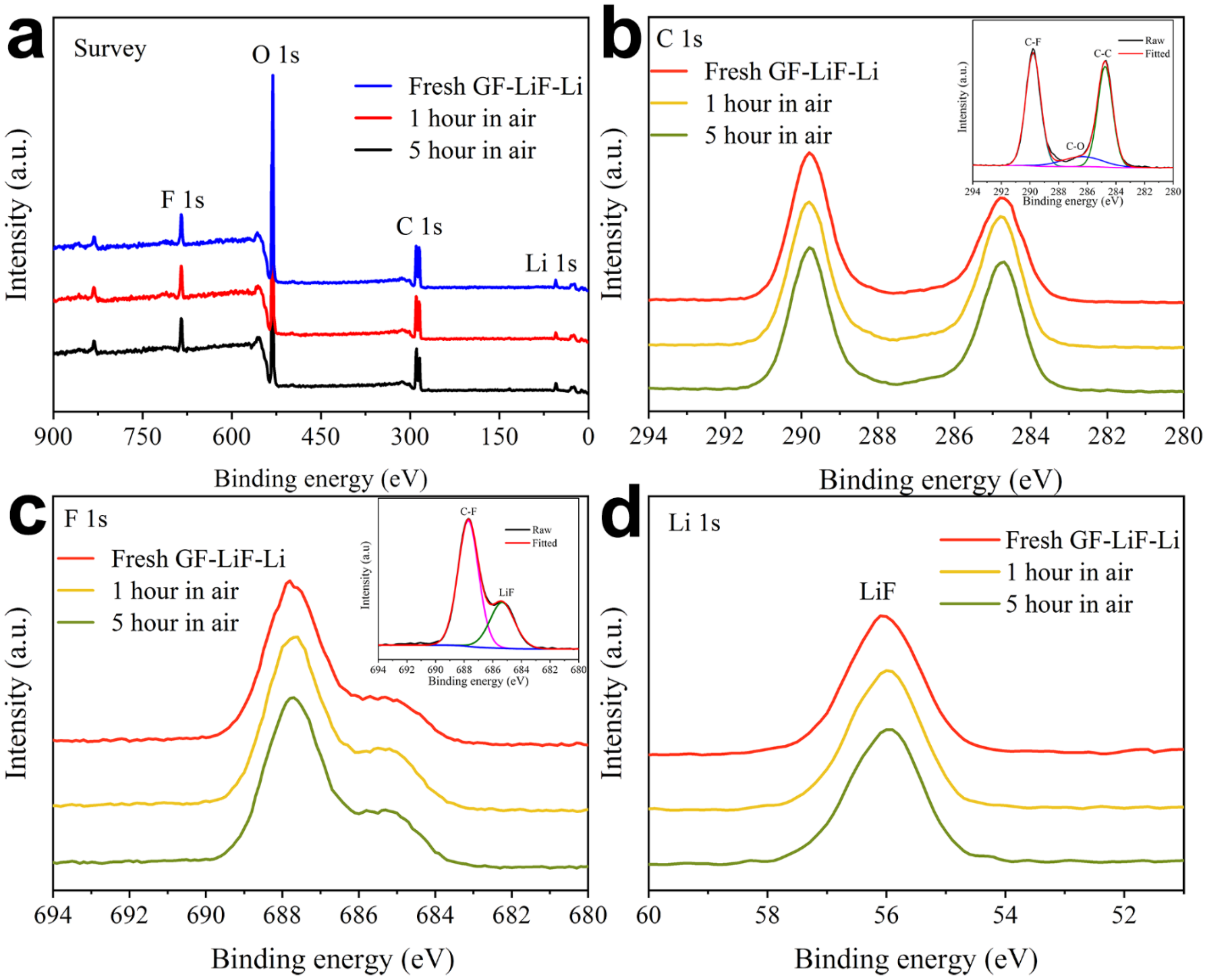

Supplementary Figure 17. XPS study of the GF-LiF-Li air-stability. **a** The survey spectra, **b** XPS C 1s, **c** F 1s, and (**d**) Li 1s spectra of GF-LiF-Li before and after 1 and 5 hours in air.

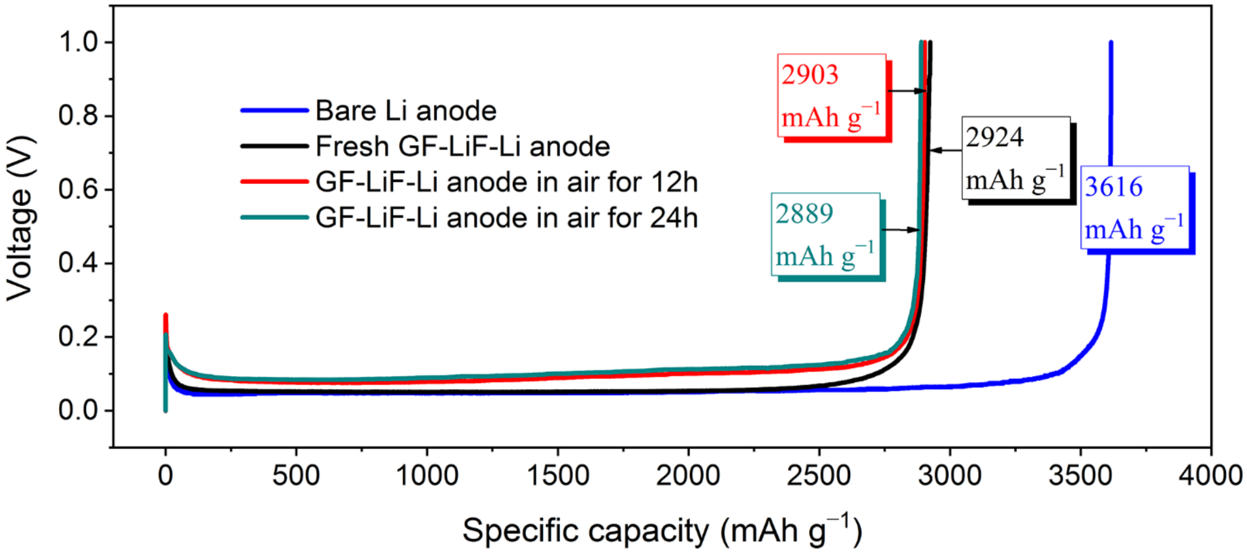

Supplementary Figure 18. The specific capacity of the bare Li anode, as well as GF-LiF-Li anode before and after exposure to air for 12 and 24 hours.

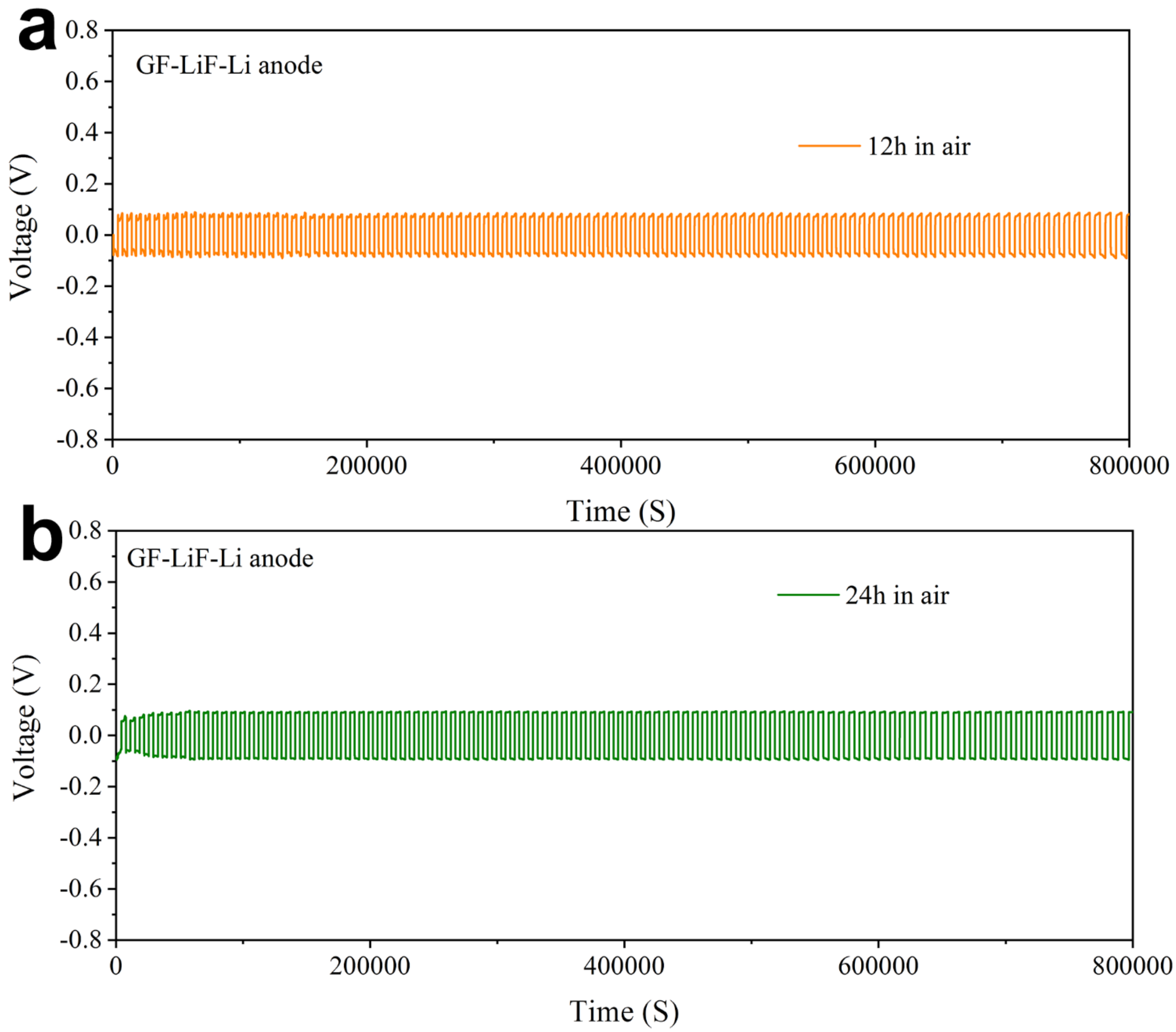

Supplementary Figure 19. Electrochemical performances of symmetrical cells with exposed GF-LiF-Li anodes. The voltage profiles of symmetrical cells with GF-LiF-Li anodes after (**a**) 12-hour and (**b**) 24-hour air exposure at a current density of 1 mA cm^{-2} for a total capacity of 1 mAh cm^{-2}.

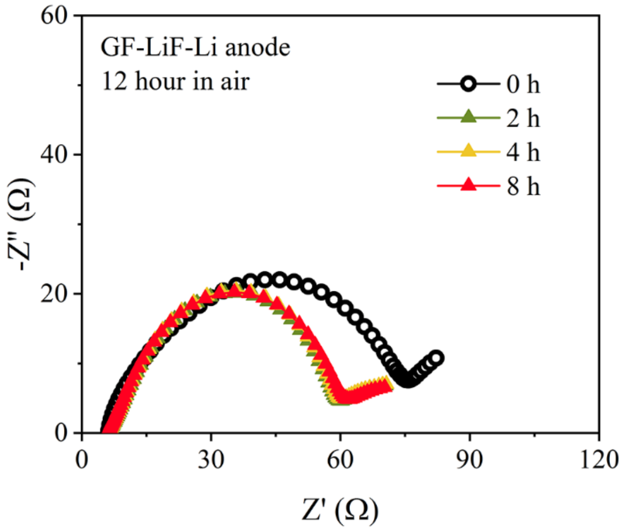

Supplementary Figure 20. EIS analysis. Time-dependent impedance spectra of symmetric batteries with GF-LiF-Li electrodes which were exposed in air for 12 hours after Li plating/string process for different durations.

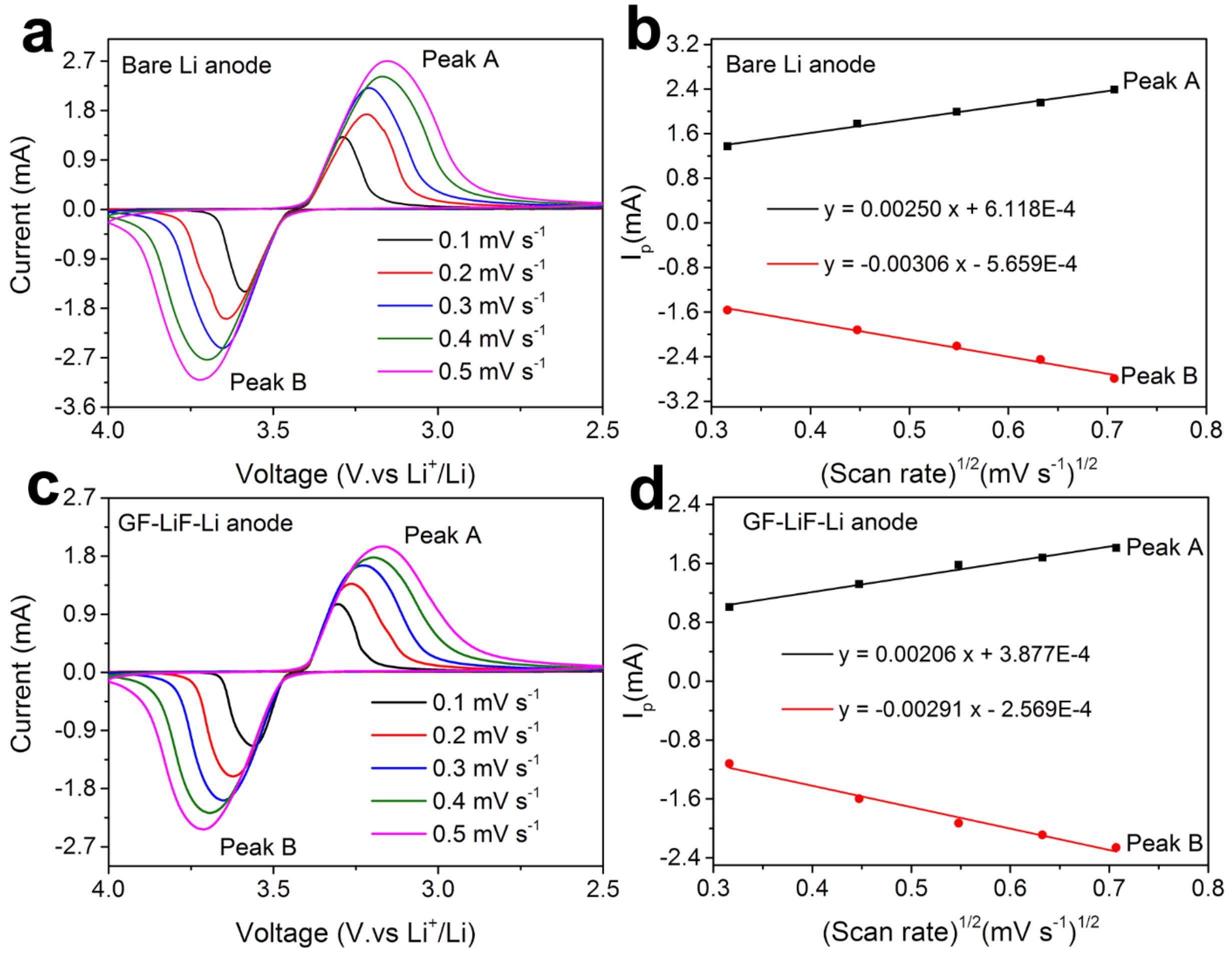

Supplementary Figure 21. Cyclic voltammograms at various voltage scan rates and corresponding linear fits of the peak currents of (**a, b**) bare Li and (**c, d**) GF-LiF-Li batteries.

Supplementary Notes

Lithium diffusion coefficient (D_{Li}) was calculate by a series of CV curves at different scan rates based on Randles-Sevcik equation: [1-2]

$$I_p = 2.69 \times 10^5 A n^{3/2} C_{Li} D_{Li}^{1/2} v^{1/2} \text{ (25 °C)} \quad (1)$$

In Supplementary Equation 1, I_p is the peak current (A), A is the area of electrode (1.13 cm^2), n is the number of reaction electrons (n=1 for Li//$LiFePO_4$ cell), C_{Li} is the concentration of Li^+ (C_{Li} = 1.0 × 10^{-3} mol cm^{-3} in (1 M $LiPF_6$/EC/DEC), v is the scanning rate, and D_{Li} is the lithium diffusion coefficient. As shown in Supplementary Fig. 13, according to the linear relationship of I_p and $v^{1/2}$, the lithium diffusion coefficients of bare Li cell are calculated to be $D_{Li\text{-}A}$ = 6.76 × 10^{-11} cm^2 s^{-1} and $D_{Li\text{-}C}$ = 1.01 × 10^{-10} cm^2 s^{-1}, and the GF-LiF-Li cells are calculated to be $D_{Li\text{-}A}$ = 4.59 × 10^{-11} cm^2 s^{-1} and $D_{Li\text{-}C}$ = 9.16 × 10^{-11} cm^2 s^{-1}.

Supplementary References

1. Chena, J., Yang, L., Fang, S., Hiranob, S., & Tachibanac, K. Synthesis of hierarchical mesoporous nest-like $Li_4Ti_5O_{12}$ for high-rate lithium ion batteries. *J. Power Sources* **200,** 59–66 (2012).
2. Ghazi, Z. A., He, X., Khattak, A. M., Khan, N. A., Liang, B., Iqbal, A., Wang, J., Sin, H., Li, L., & Tang, Z. MoS_2/celgard separator as efficient polysulfide barrier for long-life lithium-sulfur batteries. *Adv. Mater.* **29,** 1606817 (2017).
