# Supplementary material for: Lithium anode stable in air for low-cost fabrication of a dendrite-free lithium battery
Source: Nat Commun. 2019 Feb 22;10:900. doi: 10.1038/s41467-019-08767-0 (PMC6385276; doi:10.1038/s41467-019-08767-0)
Supplement: Supplementary file 2 — Description of Additional Supplementary Files [file 41467_2019_8767_MOESM2_ESM.pdf]

Description of Additional Supplementary Files

Supplementary Movie 1

Li deposition process of bare Li electrode, in symmetric cuvette-type optical cell through in-situ optical microscopy.

Supplementary Movie 2

Li deposition process of GF-LiF-Li electrode, in symmetric cuvette-type optical cell through in-situ optical microscopy.
